# Phytochemicals: Dietary Sources, Innovative Extraction, and Health Benefits

**DOI:** 10.3390/foods11010072

**Published:** 2021-12-29

**Authors:** Yolanda Aguilera, Vanesa Benítez

**Affiliations:** 1 Institute of Food Science Research, CIAL (UAM-CSIC), 28049 Madrid, Spain; 2 Department of Agricultural Chemistry and Food Science, Universidad Autónoma de Madrid, 28049 Madrid, Spain

Plants are the main natural source of numerous phytochemicals, although only a certain amount have been isolated and identified. Nutritional epidemiology has investigated the relation between diet and human health, reporting positive evidence on the role of phytochemicals. The studies carried out to date affirm that these compounds can reduce the incidence of several chronic diseases, including cardiovascular, obesity, diabetes, and cancer diseases, as well as high blood pressure and inflammation. Vegetables, fruits, pulses, chocolate, and teas are rich sources of phytochemicals; however, the wide diversity of these compounds requires optimized extraction methodologies to further characterization.

This Special Issue addresses interdisciplinary research about phytochemicals, highlighting their dietary sources, innovative extraction methodologies and their effect on human health. Seven papers have been selected to contribute further to phytochemical studies. Guava (*Psidium guajava* L.) leaves are studied by Kumar et al. [1] due to their health benefits. In this review, the authors critically discussed the presence of several bioactive compounds and the biological activities of guava leaf extracts, emphasizing the potential use of this plant’s leaves as an ingredient in the development of functional foods and pharmaceuticals. The authors reported the existence of a high level of antioxidants and phenolic compounds responsible of biological activities, including antioxidant, hypoglycemic, anticancer, antimicrobial, and anti-obesity activities. The addition of guava leaves did not cause any change in the texture properties, did not alter the rheological and sensory properties, and showed no interaction with medicines or drugs.

Two papers about legumes have been included in this Special Issue due to the importance of this food product as source of bioactive compounds. On the one hand, the study performed by Pedrosa et al. [2] is focused on the changes produced in the bioactive phytochemical content of pulses processed by domestically or industrially processing techniques to develop new pulse flour ingredients. The review describes the processing effects on bioactive phytochemicals, concluding reductions in galactosides, phenolic compounds, protein inhibitors, and myo-inositol content. Contrarily, protein digestibility and mineral absorption was improved in processed samples. This studied food-processing technology focused on the use of autoclaving, extrusion/cooking, and cold extrusion that would allow the inclusion of pulse flours in new food products. On the other hand, Moreno et al. [3] investigated the anti-adipogenesis potential of selected legume protein hydrolysates and their combinations by biochemical assays and in silico predictions. The protein hydrolysates showed high antioxidant activity and capacity to inhibit pancreatic lipase and HMG-CoA reductase, this capacity being higher when the hydrolysates of different legumes were combined. Therefore, the authors concluded that the combination of legume protein hydrolysates from different protein sources could synergistically enhance their anti-adipogenic potential.

Chupeerach et al. [4] carried out studies on the effect of steaming and fermentation on tea leaves, analyzing nutritive values, phenolics, antioxidant activities, and in vitro health properties. The results suggest that tea preparations play a significant role in the amount of nutrients and bioactive compounds. After the steaming process, most nutrients decreased and, after fermentation, energy, fat, sodium, potassium, and iron contents increased, while calcium and vitamins decreased. However, the total phenolic content and antioxidant activities increased significantly after steaming and fermentation. With respect to health properties, steamed tea exhibited a high inhibition against lipase, α-amylase, and α-glucosidase, while fermented tea possessed high anti-cholinesterases and anti-angiotensin-converting enzyme activities. Therefore, these results indicate that tea preparations could be used as a source of nutrients and bioactive compounds to develop other functional foods and drinks.

Anthocyanins, apart from their health promoting effects, are being intensely studied as natural colors. These compounds can change their structure due to chemical and enzymatic reactions that can lead to the loss of color and its beneficial properties. In this regard, Slavu et al. [5] have studied the thermal degradation kinetics of anthocyanins extracted from purple maize flour and the impact of heating on antioxidant activity and anthocyanin release during in vitro digestion. The results showed the thermostability of anthocyanins between 80 and 120 °C, following a first-order kinetic model at higher temperatures. The thermal degradation of anthocyanins reduced their antioxidant activity, and heating decreased their stability and release after in vitro digestion.

The Special Issue also incorporates a study about the revalorization of industry by-products, in particular coffee husks, by optimizing the extraction of phenolic compounds using green sustainable techniques. Rebollo-Hernanz et al. [6] used artificial neural networks and response surface methodology to model the effect of time, temperature, acidity, and the solid-to-liquid ratio on phenolic compounds. The results validated the model and the phenolic aqueous extract obtained (100 °C, 90 min, no acid, 0.02 g of coffee husk per mL) could be utilized as nutraceutical or sustainable food ingredient.

Finally, a preliminary verification of the safety of a European hybrid of *Ilex* for use as a substitute for yerba mate is included in this Special Issue. Kuropka et al. [7] studied the effect of *Ilex x meserveae* S. Y. Hu extract and its fractions on the renal morphology of rats fed with a normal and a high cholesterol diet compared to *Ilex paraguariensis* (yerba mate). The results indicated that, at the administered concentrations, a saponin fraction from *Ilex x meserveae* seems not to influence kidney status. However, polyphenols and terpenoids existing in dry extracts and the fresh infusions from both *Ilex x meserveae* and *Ilex paraguariensis,* together with co-extracted compounds in a normal diet, cause a nephrotoxic effect that is less pronounced when a high-cholesterol diet is administered.

We are pleased to present this Special Issue that contributes to the growth of this research area. We would like to thank all the authors who have shared their scientific knowledge through their contributions and the reviewers of the papers published for their great contributions. We are also grateful to the editorial board members for their support during the preparation of this Special Issue. We sincerely hope that the readers will find this Special Issue motivating and informative.
